# Support persons’ preferences for the type of consultation and the format of information provided when making a cancer treatment decision

**DOI:** 10.1186/s13104-018-3552-x

**Published:** 2018-07-11

**Authors:** Anne Herrmann, Rob Sanson-Fisher, Alix Hall, Laura Wall, Nicholas Zdenkowski, Amy Waller

**Affiliations:** 1 Priority Research Centre for Health Behaviour, University of Newcastle and Hunter Medical Research Institute, Callaghan, Australia; 2 School of Medicine and Public Health, University of Newcastle, Callaghan, Australia; 3 School of Psychology, University of Newcastle, Callaghan, Australia; 4 Department of Medical Oncology, Calvary Mater Newcastle, Waratah, Australia; 5 Health Behaviour Research Group, University of Newcastle, University Drive, W4, HMRI Building, Callaghan, NSW 2308 Australia

**Keywords:** Cancer, Caregivers, Communication, Neoplasms, Oncology, Patients

## Abstract

**Objective:**

Cancer patients and their support persons commonly feel overwhelmed when being confronted with their diagnosis and treatment options. We used a DCE to examine patients’ and support persons’ preferences for: (i) attending one 40 min consultation or two 20 min consultations when making a cancer treatment decision; and for (ii) receiving additional information in written form only or in both written and online forms. Here we focus on support persons’ preferences and whether they differ from patients’ preferences.

**Results:**

159 adult medical oncology patients and 64 of their support persons took part in this study. Participants were presented with a set of hypothetical scenarios and asked to indicate their most and least preferred scenario. 92% of support persons (n = 59) completed the DCE. Most preferred to receive two consultations along with written and online information (n = 30, 51%). This was the only scenario that was chosen by statistically significantly more support persons (*p *=0.037). The proportions of patients and support persons choosing each scenario did not differ significantly from each other (*p *>0.05). Our findings suggest that when making cancer treatment decisions, clinicians should consider offering patients and support persons written and online information, combined with two shorter consultations.

## Introduction

### Support persons can provide important insights into how to improve treatment decision making

When deciding on their cancer treatment, patients commonly seek help from their partner, family and friends [1]. Patients often value their support persons’ involvement in decision making and feel more certain about their decision after consulting their support persons [2]. Most patients want their support persons to have a say about their cancer treatment decisions [3]. Some even prefer their support persons to take the lead in treatment decision making [4, 5]. However, many support persons do not understand all the information provided to them or may be psychologically unprepared to hear the patient’s prognosis and treatment options [6]. To allow patients and support persons to consider and discuss the information provided during the consultation and facilitate support person involvement in treatment decision making, it has been suggested that they should be provided with two consultations with a short time between each consultation, combined with information presented in multiple formats [7]. Our previous work indicates that patients may prefer this consultation style to receiving one longer consultation and written information only [8]. However, data is lacking on whether support persons share this view. To our knowledge, this is the first study to examine support persons’ preferences for (i) the number and length of consultations, and (ii) the format of information provided when making a cancer treatment decision; and to assess whether their preferences align with what cancer patients would prefer. Having such data can help ensure that patients and support persons receive the resources they need to make informed healthcare decisions.

### Discrete choice experiments (DCEs) to study patients’ and support persons’ preferences

DCEs are a methodologically robust approach to assessing people’s preferences [9]. In a DCE, participants are presented with a set of hypothetical scenarios and asked to indicate their preferred option [10]. Compared to other methodologies used to elicit people’s preferences, DCEs have a number of advantages, including: reduced participant burden as they are only required to consider one single survey item, and elimination of yes-response bias as participants are forced to elicit a preference [11, 12]. There is evidence to support the internal validity and consistency of DCE designs [10]. DCEs have been used across a number of fields, including cancer research. For example, Hol et al. employed a DCE to determine, among people with an average risk of developing colorectal cancer, their preferences for various colorectal cancer screening tests [13]. Sculpher and colleagues used a DCE to establish which prostate cancer treatment attributes are most important to men [14]. DCE designs have been found a valid and reliable approach to elicit patients’ preferences for different aspects of cancer care [15, 16].

## Main text

### Aims

To examine, in a sample of cancer patient support persons, their preferences for:

i. Attending either one 40 min consultation or two 20 min consultations when making a cancer treatment decision for themselves; and
ii. The format of information they would receive regarding their treatment options (written vs written and online).

We then compared support persons’ preferences to patients’ preferences.

### Design

This was a cross-sectional study which included a DCE. Consenting participants completed a paper-and-pen survey via their preferred method (mailed or via email) within 1 week after recruitment (baseline) and 3 months later (follow-up). The DCE assessed in this study was included in the follow-up survey. Patient recruitment, data collection and patients’ preferences have been described in detail in a separate paper [8]. Here we are looking at support persons’ preferences and whether they differ from patients’ preferences. Consenting patients were asked to nominate a support person. If this person accompanied the patient to their appointment, they were approached for consent in the clinic. If the support person was not present in the clinic, consenting patients were provided with a recruitment package which included a study information letter and a survey to pass on to the eligible person.

### Sample and setting

This study was undertaken in two medical oncology treatment centres in NSW, Australia. Eligible support persons were: (i) nominated by the patient as someone helping them cope with their cancer through support, encouragement and communication; (ii) aged 18 years or over; and (iii) English speaking. Clinic staff recorded the age and gender of non-consenters who provided permission, which allowed for examination of consent bias.

### Measures

#### DCE to examine preferences for consultation type and format of information

**Fig. 1:**
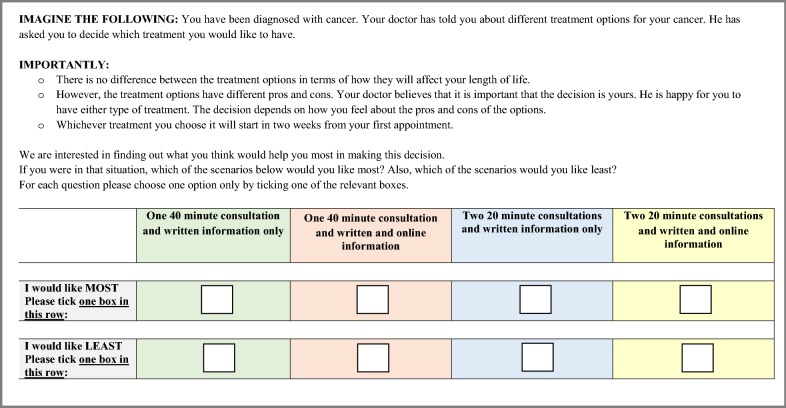
An example of the scenarios support persons could choose from to indicate their most and least preferred consultation type and format of information

The DCE consisted of two attributes with two levels each. Thus, participants were presented with four scenarios (see Fig. 1, [8]). Attributes and levels were based on a literature review and discussions among the research team. The scenarios were shown in a randomly selected order. The DCE was pilot tested with health behaviour researchers, clinicians, statisticians and cancer patients.

#### Sociodemographic characteristics

The following self-reported sociodemographic characteristics of support persons were evaluated: age, sex, relationship to patient, whether support persons were living with the patient and the time spent with the patient.

### Statistical analysis

All analyses were conducted in Stata 14.2 and R 3.4.0 (2017-04-21). Consent bias with regard to sex and age were assessed using Chi squared tests. The DCE data was analysed using descriptive statistics, Pearson’s Chi squared test with Yates’ continuity correction and an ordinal regression model. This enabled us to examine the trade-offs participants made when choosing between the different levels of the attributes. Chi squared tests were used to examine if the proportions of support persons who chose each scenario were statistically significantly different from the proportions of patients choosing each scenario, using a *p* value cut-off of 0.05. Bootstrapping was used to calculate 95% confidence intervals.

### Results

#### Participants

One hundred thirteen support persons filled out the baseline survey, of which 74% (n = 84) consented to be sent a follow-up survey. Of these, 64 (76%) completed the questionnaire. There were no statistically significant differences between consenters and non-consenters in terms of age and gender (p > 0.05). Support persons had a mean age of 61 years. Most support persons were female (n = 41, 64%) and reported to be the patient’s spouse or partner (n = 37, 58%, see Table 1). Patients’ consent and response rates as well as patient characteristics have been described in detail elsewhere [8].

**Table 1:** Sociodemographic characteristics of support persons

	Respondents n = 64 (%)
Age in years, mean (SD)	61 (13)
Gender	
Male	23 (36)
Female	41 (64)
Relationship to the patient	
Spouse/partner	37 (58)
Relative	24 (38)
Other	3 (4.6)
Living with the patient	
Yes	42 (66)
No	22 (34)
Time spent caring for patient	
Less than 20 h	31 (48)
20–40 h	10 (16)
More than 40 h	10 (16)
Unsure or do not provide any care	11 (17)
Missing	2 (3.1)

#### Support persons’ preferences

**Fig. 2:**
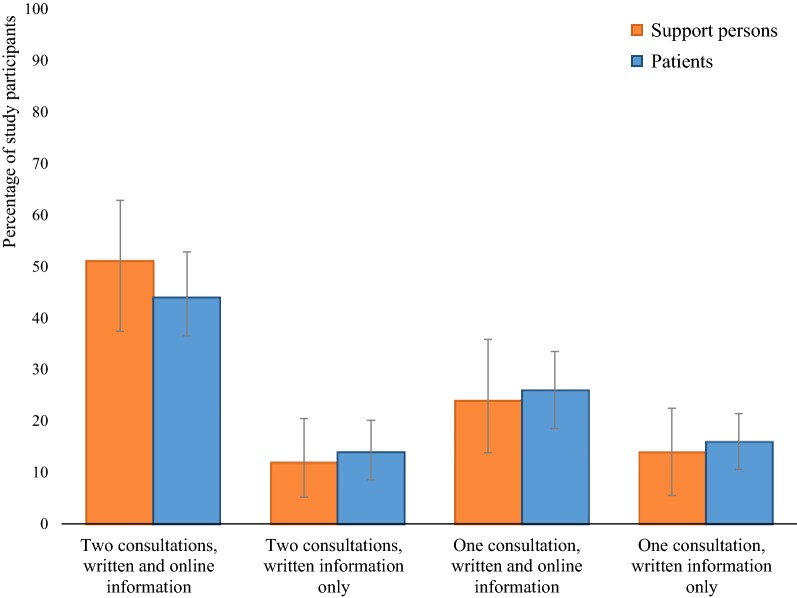
Patients’ and support persons’ preferences for scenarios

Ninety-two percent of support persons (n = 59) completed the DCE. Just over half of support persons (n = 30, 51%) preferred to receive two consultations combined with written and online information when making a cancer treatment decision for themselves (see Fig. 2). The second most preferred scenario included one consultation and written and online information, with 24% of support persons (n = 14) preferring this option. The third most preferred scenario included one consultation and written information only (14%, n = 8). Support persons preferred least to receive two consultations and written information only (12%, n = 7). The only scenario that was chosen by statistically significantly more support persons included two consultations and written and online information (p = 0.037). The percentages of support persons choosing one of the other scenarios did not differ significantly from each other.

#### Patients’ preferences

Cancer patients’ preferences are presented in detail in a separate paper [8]. Of the 147 patients completing the DCE, most preferred to receive two consultations along with written and online information (n = 65; 44%, see Fig. 2). Statistically significantly more patients preferred to receive two shorter consultations over one longer consultation, when this was combined with being provided with additional written and online information (p < 0.01).

#### Comparing patients’ and support persons’ preferences

The proportions of support persons choosing each scenario did not differ statistically significantly from patients’ preferences (p > 0.05, see Fig. 2).

### Discussion

We examined support persons’ preferences for different characteristics of oncology consultations when making a cancer treatment decision, and whether these preferences differed to what patients preferred. Our data indicate that most support persons would prefer to receive two shorter consultations and both written and online information when deciding on their treatment, which was also true for patients. We found no difference in the proportions of support persons’ and patients’ preferences for the other options. Both patients and support persons seem to be driven by the same preferences for how to make cancer treatment decisions. They appear to prefer to receive information on the available treatment options in multiple formats and would like to have two consultations to make the decision [8]. This might facilitate a shared approach towards decision making between patients, their support persons and treating clinicians by allowing patients and support persons to talk about the information provided by their doctors. Also, receiving information via multiple channels might help patients and support persons access information according to their individual preferences and assist them with comprehending, weighing-up and using the information presented to them during the consultation [17].

#### Why incorporating patients’ and support persons’ preferences for how to make treatment decisions

In order to be patient-centred, healthcare needs to align with patients’ preferences and incorporate sociocultural influences, such as support persons’ needs and wishes [18]. Our data suggest that patients and their support persons may have similar views about how to make cancer treatment decisions. As such, support persons may be a source of information about patients’ wishes which could help doctors identify patients’ decision making preferences and tailor care accordingly. Also, the importance of support persons for patients’ decision making process has been highlighted by a number of health psychology theories, such as the Theory of Reasoned Action and the Theory of Planned Behaviour [19, 20]. These theories suggest that deciding on patient care can be influenced by the so-called “subjective norm” which refers to i) what beliefs the patients think that their support persons hold about the decision at hand, and ii) the extent to which patients are influenced by these others [19, 20]. Clinicians need to be aware of support persons’ role in the decision making process when aiming to support patients with making treatment decisions. Aligning care with patients’ and support persons’ wishes can improve patient outcomes, for example by reducing conflicts between doctors, patients and support persons [21]. It can further improve patients’ recovery from their discomfort and concern, improve their emotional wellbeing and treatment adherence, and ultimately lead to more efficient and effective patient care [22, 23].

#### Conclusions

Support persons can play an important role in treatment decision making and their preferences need to be taken into account in order to achieve optimal, patient-centred cancer care [23, 24]. Based on our findings, patients and support persons seem to prefer the idea of having two shorter consultations supplemented with written and online information, rather than one longer consultation and written information alone when making cancer treatment decisions. Offering this consultation style to patients might help involve their support persons in the decision making process and assist patients with making informed decisions regarding their care. Intervention studies are needed to examine how different consultation styles may impact on patients’ and support persons’ outcomes.

## Limitations

It has been argued that people’s preferences for choosing hypothetical scenarios may differ from their preferences for making actual decisions [25]. However, several studies have compared actual choices with stated preferences and found that parameters from both were similar [26]. Also, we examined support persons’ preferences with regard to what they would want if they decided on their own cancer treatment. However, they may not have experienced cancer themselves. Consequently, their answer may not reflect what they would prefer if they were faced with this decision. Finally, support persons’ preferences for what they would want for themselves may differ from what they would choose when supporting the patient they care for. However, we were interested in how support persons would prefer to make treatment decisions to better understand how we could help them “digest” the provided information and become involved in treatment decision making. Also, it has been suggested that support persons’ views on how they prefer to make cancer treatment decisions can impact on the relationship between doctor and patient [21]. Thus, it is important to study support persons’ views on what they would choose if they had to decide on their own treatment.

## Abbreviations

DCE: discrete choice experiment
NSW: New South Wales
